# Retraction: The effects of the M2a macrophage-induced axonal regeneration of neurons by arginase 1

**DOI:** 10.1042/BSR20193031_RET

**Published:** 2026-08-14

**Authors:** Jieyuan Zhang, Yue Li, Zhaoxia Duan, Jianyi Kang, Kuijun Chen, Guanhua Li, Changmei Weng, Dongdong Zhang, Lu Zhang, Jianmin Wang, Bingcang Li

**Affiliations:** 1 Daping Hospital, Army Medical University, State Key Laboratory of Trauma, Burn and Combined Injury, 400042 Chongqing, People's Republic of China; 2 Xinan Hospital, Army Medical University, 400038 Chongqing, People's Republic of China; 3 Department of Pediatric Research Institute, Children's Hospital of Chongqing Medical University, Ministry of Education Key Laboratory of Child Development and Disorders, China International Science and Technology Cooperation Base of Child Development and Critical Disorders, Chongqing Key Laboratory of Translational Medical Research in Cognitive Development and Learning and Memory Disorders, Chongqing 400014, China

**Keywords:** Arg1, Cdc42/N-WASP, M2a, macrophage, Spinal cord injury

This article is being retracted from *Bioscience Reports* at the request of the Editor-in-Chief and the Editorial Board.

This follows the receipt of a notification from a reader, alerting the Editorial Board to an instance of high similarity in Figure 1A between the RAW264.7/0 h and RAW264.7 + IL4/0 h panels.

Upon investigation, a second instance of high similarity was noted between the Figure 2A B-actin bands and the Figure 2B GAPDH bands (lanes 1-3) from Li et al. 2019 (DOI: 10.1042/BSR20190749). There are additional concerns surrounding authorship and potential peer review manipulation.

The authors were contacted with regard to the retraction and did not respond to the Journal's queries or the concerns raised. Given the extent of the issues raised, the Editorial Board stands by the decision to retract the article.

